# *Spirulina platensis* as a novel natural antimicrobial against macrolide-resistant *Mycoplasma gallisepticum* in poultry

**DOI:** 10.3389/fmicb.2026.1769010

**Published:** 2026-02-18

**Authors:** Sabrina Zidi, Nadine Khadraoui, Rym Essid, Imen Chniba, Sarra Abassi, Salim Chibani, Maher Gazbar, Behija Mlik, Hédi Gazbar, Wassim Y. Almawi, Boutheina Ben Abdelmoumen Mardassi

**Affiliations:** 1 Group of Mycoplasmas, Laboratory of Molecular Microbiology, Vaccinology, and Biotechnology Development, Pasteur Institute of Tunis, University of Tunis El Manar, Tunis, Tunisia; 2 Laboratory of Medical Parasitology, Biotechnology and Biomolecules, Pasteur Institute of Tunis, University of Tunis El Manar, Tunis, Tunisia; 3 Laboratory of Bioactive Substances, Center of Biotechnology of Borj Cedria, Tunis, Tunisia; 4 Eden Life Industry, Gabès, Tunisia; 5 Faculty of Sciences of Tunis, University of Tunis El Manar, Tunis, Tunisia

**Keywords:** antimicrobial resistance, avian, broth microdilution, MIC, *Mycoplasma gallisepticum*, *Spirulina platensis*

## Abstract

**Background:**

Antimicrobial resistance in *Mycoplasma gallisepticum* (MG) poses a significant threat to global poultry production, as traditional antibiotics, particularly macrolides, are becoming increasingly ineffective due to growing resistance. This study investigates *Spirulina platensis* as a natural antimicrobial candidate against macrolide-resistant MG strains.

**Materials and methods:**

We tested 64 field isolates, along with the reference strain ATCC 15302, using broth microdilution. Minimum inhibitory concentrations (MICs) were measured for conventional antibiotics (doxycycline, oxytetracycline, tylosin, tilmicosin, Aivlosin) and *Spirulina* extract. Principal Component Analysis and correlation matrices were employed to investigate therapeutic relationships, while cytotoxicity assays evaluated safety profiles.

**Results:**

High resistance rates were observed for the macrolides tilmicosin (87.5%) and tylosin (68.75%). *Spirulina platensis* showed *in vitro* antimicrobial activity against the tested isolates, with MICs ranging from 3.9 to 1,000 μg/mL; 65% of isolates were inhibited at concentrations of 250 μg/mL or lower, indicating measurable activity and supporting further investigation as a natural antimicrobial compound. Correlation analyses indicated weak or negligible associations with conventional antibiotics (*p* < 2.2 × 10^−16^), reflecting a distinct activity profile. Additionally, *Spirulina* exhibited no cytotoxicity up to 4,000 μg/mL, with a selectivity index of 512.8, indicating a favorable *in vitro* safety profile.

**Conclusion:**

These findings suggest that *Spirulina platensis* acts as a uniquely mechanistic, non-toxic antimicrobial agent that can bypass existing resistance mechanisms. Further *in vivo* validation and mechanistic investigations are necessary to assess its potential as an alternative therapeutic option for poultry health management and combating antimicrobial resistance.

## 1 Introduction

*Mycoplasma gallisepticum* (MG), one of the smallest self-replicating bacteria (0.2–0.8 μm), is a primary etiological agent of chronic respiratory disease in poultry and represents a substantial economic burden to the global poultry industry (Fang et al., 2025; Marouf et al., 2022). As a member of the class Mollicutes within the phylum Mycoplasmatota, MG lacks a peptidoglycan cell wall and instead has a cholesterol-rich, triple-layered plasma membrane, which provides resistance to β-lactams and glycopeptides (Marouf et al., 2022; Rudman et al., 2025). Its motility and pleomorphism increase its pathogenic potential (Leigh and Evans, 2024; Papazisi et al., 2003). Metabolically, MG ferments glucose and reduces tetrazolium but lacks phosphatase activity and the ability to hydrolyze arginine. Clinically, it causes respiratory illness in chickens and sinusitis in turkeys, with symptoms such as nasal discharge, facial swelling, conjunctivitis, and air sacculitis, leading to decreased feed intake, stunted growth, and productivity losses (Hamzah et al., 2022; Yadav et al., 2022).

As in other countries, the economic impact of MG is significant in Tunisia, where poultry farming accounts for most, if not all, of the country’s egg production (Groupement Interprofessionnel des Produits Avicoles et Cunicoles (GIPAC), 2021). Tetracyclines (e.g., doxycycline, oxytetracycline) and macrolides (e.g., tylosin, tilmicosin) represent the most frequently administered antibiotics for mycoplasmosis, largely due to their broad-spectrum activity and practical delivery via feed or water. However, their widespread and often intensive use has led to progressively increasing resistance worldwide, including in Tunisia, constituting a significant public health and veterinary concern (Boujemaa et al., 2020; Dégrange et al., 2008; Yin et al., 2017). The clonal spread of resistance to enrofloxacin and spiramycin by local MG strains was associated with mutations in the quinolone resistance-determining region and 23S rRNA gene (Chniba et al., 2020). This suggests that the extensive, often unwarranted use of antimicrobials facilitates the emergence of resistant MG strains, which, in turn, compromises treatment effectiveness and complicates avian health management (Kamal et al., 2025; Reinhardt et al., 2002; Patangia et al., 2022). In addition to the emergence of resistance, the misuse of antibiotics disrupts normal microbiota, weakens host immunity, and increases the risk of human exposure to drug residues (Patangia et al., 2022). This highlights the urgent need for responsible antibiotic use, alternative therapies such as phytotherapy, and tighter regulatory control (Patangia et al., 2022; Piddock et al., 2024).

Recent studies have increasingly demonstrated the potential of natural alternatives to antibiotics, with *Spirulina platensis*, a nutrient-rich blue-green microalga, emerging as a sustainable and eco-friendly option to address the growing issue of antimicrobial resistance (Woo et al., 2023). *Spirulina* is rich in bioactive compounds that support its antioxidant and immune-boosting properties, including C-phycocyanin (a complete protein containing all essential amino acids), essential fatty acids, minerals, pigments, carotenoids, flavonoids, and a wide range of vitamins (Bortolini et al., 2022; Podgórska-Kryszczuk, 2024; Salmeán et al., 2015). In addition to its documented nutritional value, *Spirulina* exhibits significant pharmacological effects, including antibacterial (Podgórska-Kryszczuk, 2024; Elshouny et al., 2021), antifungal (Gheda and Ismail, 2020), and antiviral (Gheda and Ismail, 2020; Cirne-Santos et al., 2024) activities. The therapeutic benefits of *Spirulina* also include liver protection (Mohamed et al., 2021), anticancer properties (Koníčková et al., 2014), and improved cardiovascular outcomes resulting from reduced oxidative damage and lipid peroxidation (Koite et al., 2022). Collectively, this positions *Spirulina platensis* as a viable supplement in poultry health management and a promising candidate for antibiotic-free strategies in sustainable animal production systems (Bortolini et al., 2022; El-Shall et al., 2023).

Owing to its immune-boosting properties, earlier studies have demonstrated the efficacy of *Spirulina* in managing several poultry diseases, including Newcastle disease and other viral infections, via the *Spirulina* polysaccharide, which enhances macrophage cytokine and nitric oxide production (Abotaleb et al., 2020; Abdo et al., 2019). Its antibacterial potential is primarily attributed to active constituents such as polysaccharides and phycocyanin, which disrupt bacterial cell membrane integrity, induce oxidative stress, and chelate essential metals necessary for bacterial metabolism and survival (Woo et al., 2023; Bortolini et al., 2022). The objective of this study was to assess the *in vitro* antimicrobial activity of *Spirulina platensis* against antibiotic-resistant *Mycoplasma gallisepticum* strains and explore its potential as a complementary natural compound in poultry health management.

## 2 Materials and methods

### 2.1 Mycoplasma strains

Between 2022 and 2025, 65 MG strains isolated from commercial broiler and layer flocks exhibiting clinical symptoms consistent with Mycoplasma infection across various regions of Tunisia were analyzed in this study (Table 1). Cultivation was performed in Frey’s broth, which was enriched with heat-inactivated fetal bovine serum, L-arginine, beta-nicotinamide adenine dinucleotide (*β*-NAD), phenol red (as a pH indicator), yeast extract, and glucose. To reduce bacterial contamination, penicillin was added to the medium. Inoculated cultures were incubated at 37 °C under microaerophilic and humidified conditions. Bacterial growth was assessed by changes in pH and media turbidity. Three successive rounds of cloning from individual colonies were conducted to ensure the purity of the isolates and genetic uniformity. Molecular identification of MG strains was confirmed by PCR using primers specific to the pMGA gene, a reliable genetic marker for MG, as described elsewhere (Mardassi et al., 2005).

**Table 1:** Characteristics and metadata of 65 *M. gallisepticum* strains.

Isolates ID	Year of isolation	Host of isolation	Age of the host	Antibiotic treatment history	
				Antibiotic names	Post-antibiotic sampling interval
S6 ATCC15302	-				
MG1/24	2024	Turkey	109 days	Doxycycline, tilmicosin	25 days
MG2/24	2024	Turkey	109 days	Doxycycline, tilmicosin	25 days
MG3/24	2024	Turkey	109 days	Doxycycline, tilmicosin	25 days
MG4/24	2024	Turkey	109 days	Doxycycline, tilmicosin	25 days
MG5/24	2024	Turkey	109 days	Doxycycline, tilmicosin	25 days
MG6/24	2024	Turkey	109 days	Doxycycline, tilmicosin	25 days
MG7/24	2024	Turkey	109 days	Doxycycline, tilmicosin	25 days
MG8/24	2024	Turkey	109 days	Doxycycline, tilmicosin	25 days
MG9/24	2024	Turkey	109 days	Doxycycline, tilmicosin	30 days
MG10/24	2024	Turkey	109 days	Doxycycline, tilmicosin	25 days
MG11/24	2024	Turkey	109 days	Doxycycline, tilmicosin	25 days
MG12/24	2024	Turkey	109 days	Doxycycline, tilmicosin	25 days
MG13/24	2024	Turkey	109 days	Doxycycline, tilmicosin	25 days
MG14/24	2024	Turkey	109 days	Doxycycline, tilmicosin	30 days
MG15/24	2024	Turkey	109 days	Doxycycline, tilmicosin	25 days
MG16/24	2024	Turkey	109 days	Doxycycline, tilmicosin	25 days
MG17/24	2024	Turkey	109 days	Doxycycline, tilmicosin	25 days
MG18/24	2024	Turkey	109 days	Doxycycline, tilmicosin	31 days
MG19/24	2024	Turkey	109 days	Doxycycline, tilmicosin	25 days
MG20/24	2024	Turkey	109 days	Doxycycline, tilmicosin	25 days
MG21/24	2024	Turkey	109 days	Doxycycline, tilmicosin	25 days
MG22/24	2024	Turkey	109 days	Doxycycline, tilmicosin	25 days
MG23/24	2024	Turkey	109 days	Doxycycline, tilmicosin	25 days
MG24/24	2024	Turkey	109 days	Doxycycline, tilmicosin	25 days
MG25/24	2024	Turkey	109 days	Doxycycline, tilmicosin	25 days
MG26/24	2024	Turkey	109 days	Doxycycline, tilmicosin	25 days
MG27/24	2024	Turkey	109 days	Doxycycline, tilmicosin	25 days
MG28/24	2024	Turkey	109 days	Doxycycline, tilmicosin	25 days
MG29/24	2024	Turkey	109 days	Doxycycline, tilmicosin	25 days
MG30/24	2024	Turkey	109 days	Doxycycline, tilmicosin	25 days
MG31/24	2024	Turkey	109 days	Doxycycline, tilmicosin	25 days
MG32/24	2024	Turkey	109 days	Doxycycline, tilmicosin	25 days
MG33/24	2024	Turkey	109 days	Doxycycline, tilmicosin	25 days
MG34/24	2024	Turkey	109 days	Doxycycline, tilmicosin	21 days
MG35/24	2024	Turkey	109 days	Doxycycline, tilmicosin	25 days
MG36/24	2024	Turkey	109 days	Doxycycline, tilmicosin	25 days
MG37/24	2024	Turkey	109 days	Doxycycline, tilmicosin	25 days
MG38/24	2024	Turkey	108 days	Doxycycline, tilmicosin	25 days
MG39/24	2024	Turkey	108 days	Doxycycline, tilmicosin	25 days
MG40/24	2024	Turkey	108 days	Doxycycline, tilmicosin	21 days
MG41/24	2024	Turkey	108 days	Doxycycline, tilmicosin	25 days
MG42/24	2024	Turkey	108 days	Doxycycline, tilmicosin	25 days
MG43/24	2024	Turkey	108 days	Doxycycline, tilmicosin	25 days
MG44/24	2024	Turkey	108 days	Doxycycline, tilmicosin	25 days
MG45/24	2024	Turkey	108 days	Doxycycline, tilmicosin	25 days
MG46/24	2024	Turkey	108 days	Doxycycline, tilmicosin	25 days
MG47/24	2024	Turkey	108 days	Doxycycline, tilmicosin	25 days
MG48/24	2024	Turkey	108 days	Doxycycline, tilmicosin	25 days
MG49/24	2024	Turkey	108 days	Doxycycline, tilmicosin	25 days
MG50/24	2024	Turkey	108 days	Doxycycline, tilmicosin	25 days
MG51/24	2024	Turkey	108 days	Doxycycline, tilmicosin	25 days
MG52/24	2024	Turkey	108 days	Doxycycline, tilmicosin	25 days
MG53/24	2024	Turkey	108 days	Doxycycline, tilmicosin	25 days
MG54/24	2024	Turkey	108 days	Doxycycline, tilmicosin	25 days
MG55/24	2022	Chicken	108 days	Doxycycline, tilmicosin	25 days
MG56/24	2022	Chicken	108 days	Aivlosin, doxycycline, tilmicosin	25 days
MG57/24	2022	Chicken	109 days	Aivlosin, doxycycline, tilmicosin	25 days
MG58/24	2022	Chicken	109 days	Aivlosin, doxycycline, tilmicosin	25 days
MG59/24	2022	Chicken	109 days	Aivlosin, doxycycline, tilmicosin	25 days
MG60/24	2022	Chicken	109 days	Aivlosin, doxycycline, tilmicosin	25 days
MG61/24	2022	Chicken	108 days	Aivlosin, doxycycline, tilmicosin	25 days
MG62/24	2022	Chicken	109 days	Aivlosin, doxycycline, tilmicosin	25 days
MG63/24	2022	Chicken	109 days	Aivlosin, doxycycline, tilmicosin	25 days
MG64/24	2022	Chicken	90 days	Aivlosin, doxycycline, tilmicosin	25 days

Metadata of the 65 *Mycoplasma gallisepticum* strains included in this study. The table lists each strain’s identification number (ID), host of isolation, age of the host, year of isolation and available information regarding the antibiotic treatment.

### 2.2 Source and preparation of *Spirulina* biomass

The *Spirulina platensis* used in this study was obtained from a monitored aquaculture facility operated by Eden Life,^1^ located in the Kettana oasis between Gabès and Mareth (Southern Tunisia) and situated in an ecologically protected zone, free from industrial and urban contaminants. Characterized by an arid climate, abundant sunlight, and stable temperatures, the local environment creates favorable conditions for *Spirulina* cultivation. The microclimate provided by the surrounding palm and pomegranate trees further enhances growth stability. The cyanobacteria were cultured in freshwater without synthetic chemicals. The biomass was collected after the cultivation phase, carefully dried under controlled conditions to retain its bioactive components, and then finely milled into a powder. It was stored under appropriate conditions pending use.

### 2.3 Antimicrobial activity testing and minimum inhibitory concentration (MIC)

The antimicrobial susceptibility of MG to conventionnal antibiotics used in poultry production was evaluated, focusing on tetracyclines (doxycycline, oxytetracycline) and macrolides (tilmicosin, tylosin, Aivlosin). Stock solutions of the antibiotics were prepared and stored following the manufacturers’ instructions. Regarding the MG suspension, the bacterial titer was first determined by colony counting on solid Frey’s medium, followed by tenfold serial dilutions to obtain a final inoculum of 10^5^ CFU/mL and the susceptibility was determined by the broth microdilution method (Hannan, 2000). Briefly, 96-well microtiter plates were set up for each MG strain, with five rows containing serial two-fold dilutions of each antibiotic, ranging from 32 to 0.03 μg/mL, in Frey’s broth medium and a bacterial suspension (10^5^ CFU/mL) was added to each well. The remaining three rows served as controls: a growth control without antibiotic, a sterility control with no inoculum, and a quality control with the reference strain, MG S6 (ATCC 15302). The plates were incubated at 37 °C under the appropriate conditions and monitored daily for growth. The minimum inhibitory concentration (MIC) was defined as the lowest concentration that completely inhibited visible bacterial growth as described (Hannan, 2000), using interpretive criteria. All tests were performed in duplicates.

### 2.4 *In vitro* antimicrobial activity of *Spirulina platensis*

The broth microdilution method was used to determine the MIC of *Spirulina platensis* against MG (diluted to 10^5^ CFU/mL), with DMSO (100% v/v) as the solvent. *Spirulina* powder was initially prepared at concentrations of 2, 4, and 8 mg/mL. Each solution was then sonicated at 64 °C for 15 min, followed by incubation at 56 °C for 30 min to ensure complete solubilization and optimize the release of bioactive compounds. This process maximized DMSO solubility and antibacterial activity, and the resulting formulation was selected for further analysis to ensure consistent efficacy while maintaining the integrity of *Spirulina*’s active components. A starting *Spirulina* concentration of 40 mg/mL was added to the first well, and then the solution was serially diluted twofold to 1.953 μg/mL. Control wells included a growth control without *Spirulina*, a sterility control, and a solvent control (2.5% DMSO), all prepared at the same concentrations. Plates were incubated at 37 °C, and bacterial growth was assessed by observing a color change in the medium, using phenol red as a pH indicator. The MIC was recorded as having the lowest concentration of *Spirulina* that visibly inhibited MG growth. All assays were performed in duplicate. The MIC for the *Spirulina* extract was determined in duplicate for each isolate, with a third replicate performed when results were discordant (>1 two-fold dilution difference).

### 2.5 Cytotoxicity assay

The cytotoxic effect of the *Spirulina platensis* extract was evaluated on RAW 264.7 murine macrophage cells using the MTT [3-(4,5-dimethylthiazol-2-yl)-2,5-diphenyltetrazolium bromide] assay. Cells were seeded in 96-well plates at a density of 2.0 × 10^4^ cells per well (2.0 × 10^5^ cells/mL in 100 μL of complete medium) and allowed to adhere for 24 h at 37 °C in a humidified 5% CO₂ atmosphere. Serial two-fold dilutions of the extract in culture medium, ranging from 4,000 μg/mL to 125 μg/mL, were then added to the wells. After a 24-h incubation, 10 μL of MTT solution (5 mg/mL in PBS) was added to each well, resulting in a final concentration of 0.5 mg/mL. The plates were incubated for 4 h to allow formazan crystals to form. The medium was then carefully aspirated, and the formazan crystals were dissolved in 100 μL of DMSO. Absorbance was measured at 590 nm using a microplate reader, with a reference wavelength of 620–650 nm to correct for non-specific absorption.

The assay was performed in three independent biological replicates (using separate cell passages and extract preparations). For each biological replicate, all extract concentrations and controls were tested in quadruplicate technical replicates. Cell viability for each condition was calculated as the mean of the technical replicates, and this mean value was used as a single data point (*n* = 1) per biological replicate for statistical analysis. The *Spirulina* concentration that maintained macrophage viability above 90% while retaining antibacterial activity was selected for subsequent antimicrobial testing.

**Figure 1:**
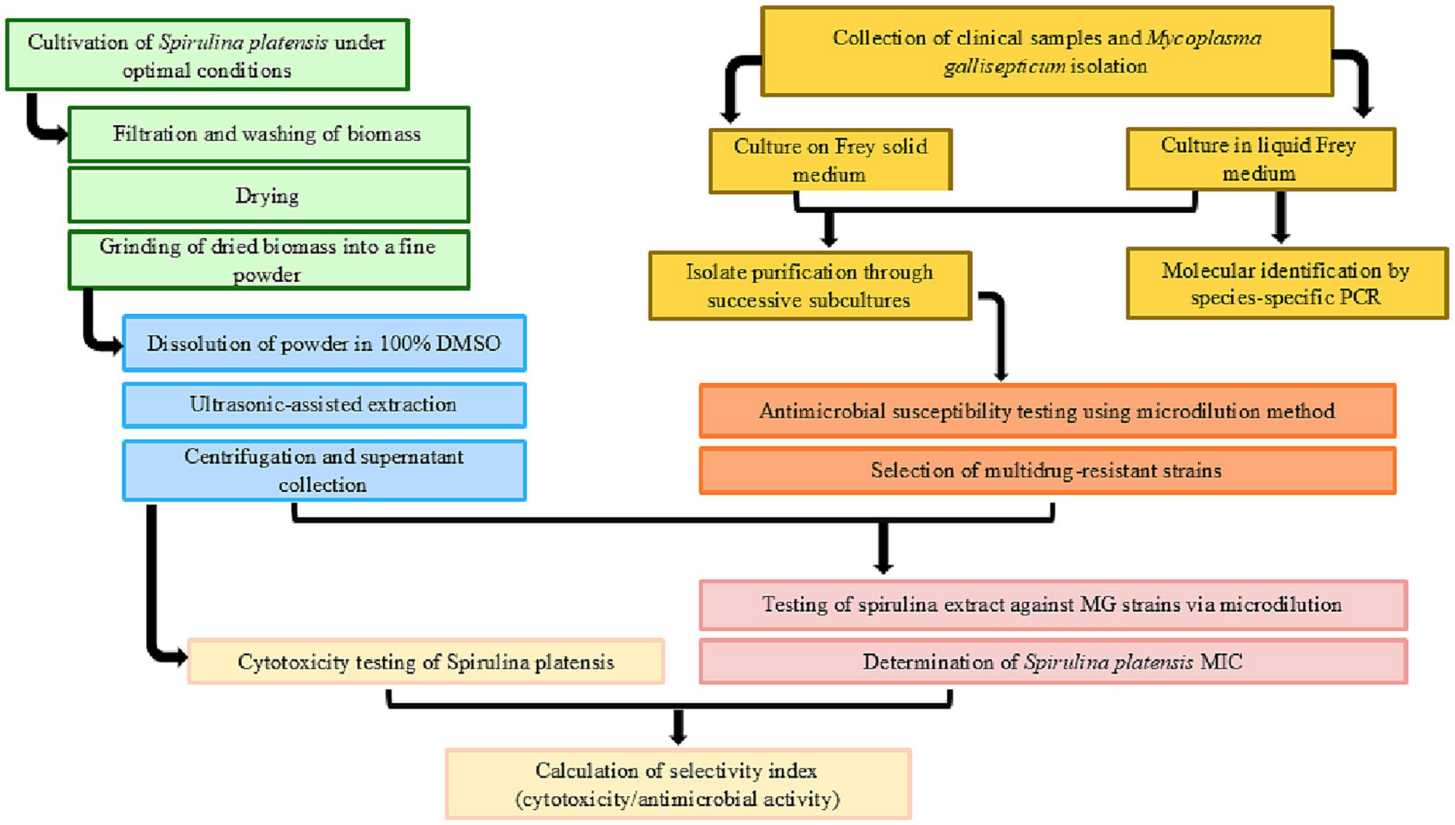
Experimental workflow: from *Spirulina platensis* cultivation to anti-mycoplasma activity. Key stages include: (1) Cultivation, harvesting, and processing of *Spirulina platensis* biomass; (2) DMSO dissolution and ultrasonic-assisted extraction; (3) Isolation and molecular identification of clinical *Mycoplasma gallisepticum* strains; (4) Antimicrobial susceptibility testing using microdilution method and selection of macrolide-resistant strains; (5) Antimicrobial susceptibility testing of *Spirulina platensis* against macrolide-resistant *M. gallisepticum* via microdilution and MIC determination and (6) Cytotoxicity assessment and selectivity index calculation.

The overall integrated methodology, from the cultivation of *Spirulina platensis* to the bioactivity assessment of its extract against *Mycoplasma gallisepticum*, is summarized in Figure 1.

### 2.6 Statistical analysis

All experiments were performed in duplicate, and the data were analyzed using RStudio (version 4.4.1). A two-way ANOVA was conducted to evaluate the effect of solvent type and concentration on the total phenolic content of *Spirulina platensis* and its antimicrobial activity. A one-way ANOVA was used to assess the other variables and to compare the effects of different concentrations of *Spirulina platensis* across various solvents with the positive controls. When results were significant (*p* < 0.05), the least significant difference (LSD) test was applied for pairwise comparisons. Pairwise associations between the log₁₀-transformed MIC values of the six antimicrobial agents were examined. After the Shapiro–Wilk test confirmed non-normal distributions for all agents (*p* < 0.05), we conducted a dual analytical approach: (1) Pearson’s product–moment correlation to quantify linear relationships, and (2) Spearman’s rank correlation as a complementary, distribution-free sensitivity analysis. This two-method strategy was implemented to ensure that any reported associations were not artifacts of distributional assumptions. Principal Component Analysis (PCA) was also performed as a multivariate approach to examine correlations between quantitative and qualitative variables. PCA reduces data complexity by transforming correlated variables into a set of uncorrelated components based on their covariance structure, enabling the identification of variables with the greatest explanatory power.

## 3 Results

### 3.1 Antibiotic susceptibility testing of MG isolates

Using the broth microdilution method, *in vitro* testing of the 64 field isolates and the ATCC reference strain revealed a wide range of MICs (Table 2). Based on established susceptibility breakpoints for MG, doxycycline demonstrated the highest efficacy, with all isolates (100%) classified as sensitive, followed closely by oxytetracycline, which exhibited a 98% sensitivity rate. On the other hand, the highest resistance levels were observed with tilmicosin (87.5%) and tylosin (68.75%). Aivlosin displayed a mixed susceptibility profile, with 60.93% of isolates categorized as intermediate, 29.68% as sensitive, and 9.37% as resistant, suggesting a shifting trend in macrolide susceptibility, including Aivlosin.

**Table 2:** Minimum inhibitory concentrations of conventional antibiotics for local MG isolates.

Isolates ID	Doxycycline (μg/mL)	S^*^	Timicosin (μg/mL)	S^*^	Tylosin (μg/mL)	S^*^	Oxytetracycline (μg/mL)	S^*^	Aivlosin (μg/mL)	S^*^
S6 ATCC15302	**0.015**	S	**0.03**	S	**0.015**	S	**0.125**	S	**0.015**	S
MG1/24	0.015	S	8	R	0.5	I	0.25	S	0.06	I
MG2/24	0.06	S	32	R	8	R	0.5	S	0.25	R
MG3/24	0.06	S	32	R	32	R	0.5	S	0.25	R
MG4/24	0.06	S	32	R	4	R	0.125	S	0.25	R
MG5/24	0.06	S	32	R	4	R	0.125	S	0.125	I
MG6/24	0.06	S	32	R	4	R	0.125	S	0.125	I
MG7/24	0.06	S	32	R	4	R	0.125	S	0.125	I
MG8/24	0.06	S	32	R	4	R	0.125	S	0.125	I
MG9/24	0.015	S	16	R	0.015	S	0.125	S	0.015	S
MG10/24	0.06	S	32	R	4	R	0.5	S	0.125	I
MG11/24	0.06	S	32	R	2	R	0.125	S	0.125	I
MG12/24	0.125	S	32	R	2	R	0.125	S	0.125	I
MG13/24	0.06	S	4	R	4	R	0.125	S	0.125	I
MG14/24	0.06	S	32	R	8	R	0.125	S	0.125	I
MG15/24	0.125	S	32	R	4	R	0.125	S	0.125	I
MG16/24	0.125	S	8	R	1	R	0.5	S	0.06	I
MG17/24	0.06	S	32	R	4	R	0.125	S	0.125	I
MG18/24	0.06	S	32	R	4	R	0.125	S	0.125	I
MG19/24	0.06	S	32	R	4	R	0.155	S	0.125	I
MG20/24	0.03	S	0.03	S	0.06	S	0.25	S	0.015	S
MG21/24	0.06	S	32	R	4	R	0.125	S	0.125	S
MG22/24	0.06	S	32	R	4	R	0.125	S	0.125	S
MG23/24	0.06	S	32	R	4	R	0.125	S	0.125	S
MG24/24	0.06	S	32	R	4	R	0.125	S	0.125	S
MG25/24	0.06	S	32	R	4	R	0.125	S	0.125	S
MG26/24	0.06	S	32	R	4	R	0.125	S	0.125	S
MG27/24	0.125	S	32	R	0.125	S	0.5	S	32	R
MG28/24	0.06	S	32	R	0.125	S	0.5	S	32	R
MG29/24	0.06	S	32	R	4	R	0.125	S	0.125	I
MG30/24	0.06	S	32	R	4	R	0.125	S	0.125	I
MG31/24	0.06	S	32	R	4	R	0.155	S	0.125	I
MG32/24	0.015	S	16	R	0.25	S	0.015	S	0.015	I
MG33/24	0.125	S	32	R	0.125	S	0.5	S	0.015	S
MG34/24	0.06	S	32	R	4	R	0.125	S	0.125	I
MG35/24	0.06	S	32	R	0.125	S	0.5	S	0.5	R
MG36/24	0.06	S	32	R	4	R	0.25	S	0.125	I
MG37/24	0.015	S	1	I	0.03	S	0.25	S	0.015	S
MG38/24	0.06	S	32	R	4	R	0.125	S	0.125	I
MG39/24	0.06	S	32	R	4	R	0.125	S	0.125	I
MG40/24	0.125	S	32	R	2	R	1	S	0.125	I
MG41/24	0.06	S	32	R	4	R	0.125	S	0.125	I
MG42/24	0.015	S	4	R	0.25	S	0.0625	S	0.015	S
MG43/24	0.06	S	32	R	4	R	0.125	S	0.125	I
MG44/24	0.06	S	32	R	2	R	0.125	S	0.125	I
MG45/24	0.125	S	16	R	2	R	1	I	0.06	I
MG46/24	0.06	S	16	R	4	R	0.25	S	0.125	I
MG47/24	0.03	S	0.03	S	0.03	S	0.125	S	0.03	S
MG48/24	0.015	S	4	R	2	R	0.25	S	0.125	I
MG49/24	0.015	S	8	R	0.5	S	0.015	S	0.015	S
MG50/24	0.015	S	0.015	S	0.5	S	0.125	S	0.125	I
MG51/24	0.015	S	8	R	0.25	S	0.0625	S	0.015	S
MG52/24	0.06	S	32	R	4	R	0.125	S	0.125	I
MG53/24	0.06	S	32	R	4	R	0.125	S	0.125	I
MG54/24	0.06	S	32	R	4	R	0.125	S	0.125	I
MG55/24	0.03	S	16	R	4	R	0.25	S	0.125	I
MG56/24	0.06	S	32	R	4	R	0.125	S	0.125	I
MG57/24	0.015	S	4	R	0.5	S	0.25	S	0.015	S
MG58/24	0.015	S	1	I	0.5	S	0.25	S	0.015	S
MG59/24	0.06	S	0.03	S	0.03	S	0.5	S	0.015	S
MG60/24	0.15	S	8	R	0.125	S	0.015	S	0.015	S
MG61/24	0.06	S	0.03	S	0.03	S	0.5	S	0.015	S
MG62/24	0.06	S	32	R	4	R	0.125	S	0.125	I
MG63/24	0.03	S	16	R	4	R	0.25	S	0.125	I
MG64/24	0.015	S	16	R	0.5	S	0.125	S	0.015	S

MG, *Mycoplasma gallisepticum*; S^*^, status; S, susceptibility; R, resistant; I, Intermediate susceptibility. *In vitro* activity of doxycycline, tilmicosin, tylosin, oxytetracycline, and Aivlosin against *Mycoplasma gallisepticum* isolates, including a reference strain (S6 ATCC15302). Values represent Minimum Inhibitory Concentration (MIC) in μg/mL, with susceptibility interpretation based on defined breakpoints (S, Susceptible; I, Intermediate; R, Resistant). The bold values correspond to the reference strain (ATCC).

### 3.2 Anti-mycoplasmal activity of *Spirulina platensis* against MG

**Figure 2:**
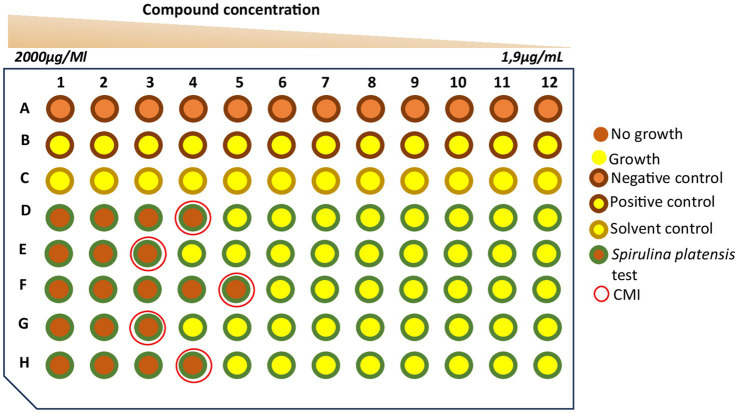
Antimicrobial activity of *Spirulina platensis* extracts against *Mycoplasma gallisepticum*. Assessment of growth inhibition against *Mycoplasma gallisepticum* (MG) in Frey medium. Two-fold serial dilutions of the extract (2000–1.9 μg/mL; rows D–H). Bacterial metabolism changes the medium from orange to yellow; inhibition prevents this change and keeps the medium orange. The minimum inhibitory concentration (MIC), defined as the lowest concentration that prevents this color change, is indicated by a red circle. Controls: (A) Positive growth control (MG + medium); (B) negative sterility control (medium only); (C) solvent control (MG + medium + solvent).

The established MIC values of *Spirulina platensis* against MG isolates, ranging from 3.9 to 1,000 μg/mL, indicated broad-spectrum antibacterial activity (Table 2). The principle of MIC determination via broth microdilution is illustrated schematically in Figure 2, which depicts the transition from growth inhibition to bacterial growth across a serial dilution of the extract. Most of the tested strains (>65%) were inhibited at concentrations of 250 μg/mL or less, with several isolates showing notable sensitivity at low MICs (3.9 μg/mL and 15.62 μg/mL). These findings emphasize the significant antimicrobial potential of *Spirulina*, particularly against strains resistant to conventional antibiotics.

### 3.3 MIC variability among antimicrobial treatments

**Figure 3:**
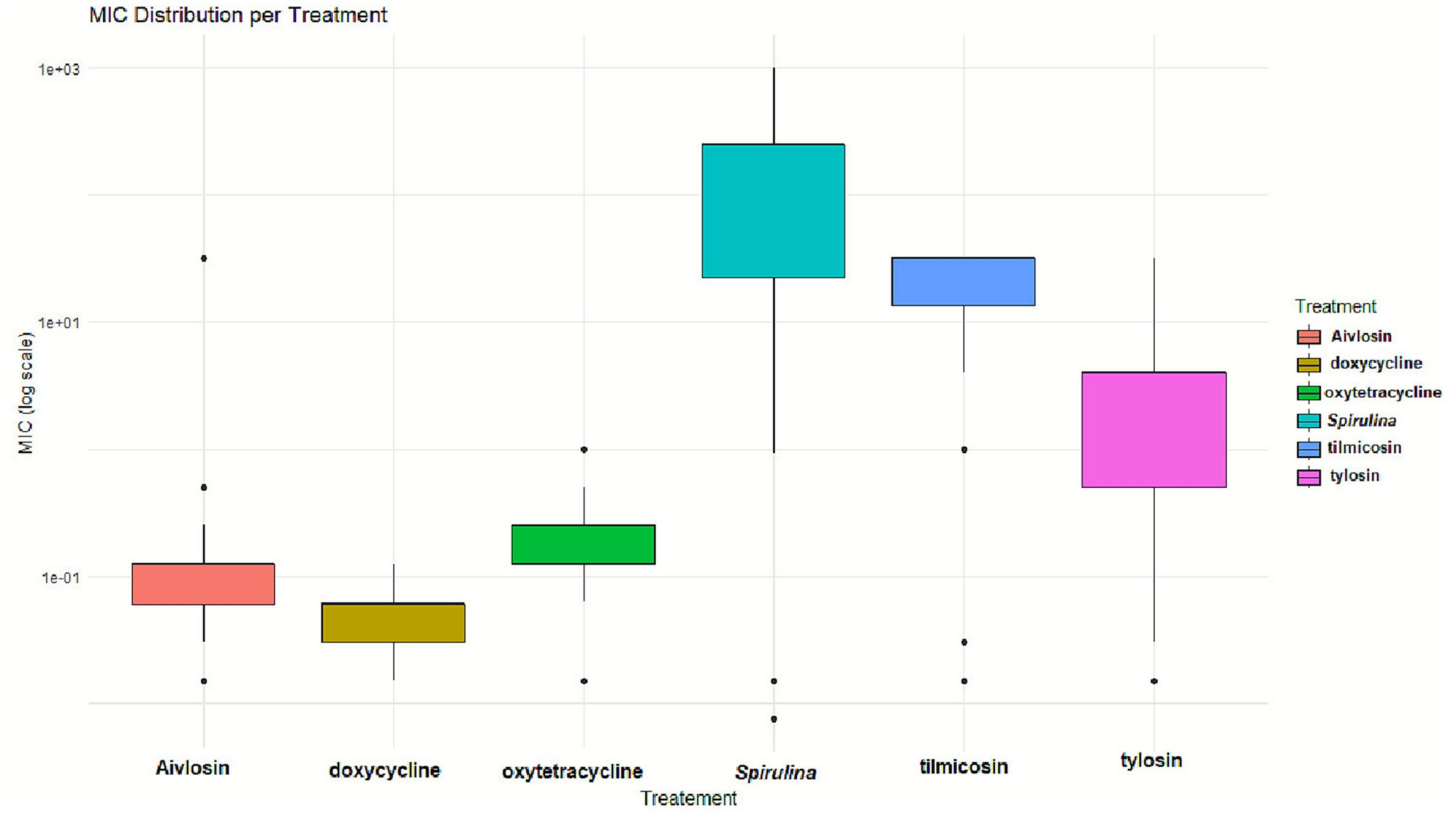
Comparative MIC distribution of antimicrobials against *MG* isolates. Box-and-whisker plots representing the MICs (μg/mL) of 65 *MG* isolates exposed to doxycycline (DOX), oxytetracycline (OXY), tilmicosin (TIL), tylosin (TYL), Aivlosin (AIV), and *Spirulina platensis* extract (SPI). Each box denotes the interquartile range with the median highlighted, whiskers mark the full data span, and individual points represent statistical outliers.

The distribution of MIC values for each treatment group was assessed using the Shapiro–Wilk test prior to comparative analysis. The results indicated that MIC values were not normally distributed across all treatments, with *p*-values < 0.05 in each case, including doxycycline (*W* = 0.757), *Spirulina platensis* (*W* = 0.678), and tylosin (*W* = 0.463), confirming significant deviation from normality. As a result, the nonparametric Kruskal-Wallis rank-sum test was used to compare MIC distributions among the six antimicrobial agents. Results from Figure 3 showed a highly significant difference in MIC values among the treatments (χ^2^ = 270.89, df = 5, *p* < 2.2 × 10^−16^), with a very large effect size (ε^2^ = 0.85, 95% CI [0.80, 0.89]). This effect size reflects the proportion of variability in MIC values explained by treatment group, without implying differences in absolute antimicrobial potency, which is indicated by the MIC magnitudes.

### 3.4 Dunn post-hoc analysis

Following the significant Kruskal-Wallis result, a Dunn’s *post hoc* test with Bonferroni correction was conducted to identify specific pairwise differences between treatments. The results, summarized in Table 3, confirmed distinct antimicrobial profiles among the agents tested.

**Table 3:** Pairwise comparisons of antimicrobial activity (MIC distributions) using the Dunn post-hoc test with Bonferroni correction.

Comparison	*Z*	*P_unadj_*	*P_adj_*	Effect size (r_rβ_)	95% CI for r_rβ_
Aivlosin - doxycycline	2.498	0.012	0.186	0.312	[0.06;0.52]
Aivlosin - oxytetracycline	−1.965	0.049	0.740	−0.246	[−0.49;0.02]
Doxycycline - oxytetracycline	−4.446	8.724 e^−06^	1.308 e^−04^	−0.556	[−0.74;-0.30]
Aivlosin - *Spirulina*	−11.032	2.675 e^−28^	4.012 e^−27^	−1.379	[−1.51;-1.21]
Doxycycline - *Spirulina*	−13.478	2.105 e^−41^	3.158 e^−40^	−1.685	[−1.80; −1.53]
Oxytetracycline - *Spirulina*	−9.031	1.693 e^−19^	2.540 e^−18^	−1.129	[−1.30; −0.92]
Aivlosin - tilmicosin	−8.799	1.374 e^−18^	2.061 e^−17^	−1.100	[−1.27; −0.89]
Doxycycline - tilmicosin	−11.263	1.982 e^−29^	2.973 e^−28^	−1.408	[−1.54; −1.23]
Oxytetracycline - tilmicosin	−6.799	1.048 e^−11^	1.572 e^−10^	−0.850	[−1.06;-0.60]
*Spirulina* - tilmicosin	2.267	0.023	0.350	0.283	[0.03;0.50]
Aivlosin - tylosin	−5.196	2.028 e^−07^	3.042 e^−06^	−0.650	[−0.87, −0.38]
Doxycycline - tylosin	−7.675	1.652 e^−14^	2.478 e^−13^	−0.959	[−1.16; −0.71]
oxytetracycline - tylosin	−3.211	0.0013	0.019	−0.401	[−0.66; −0.10]
*Spirulina* – tylosin	5.855	4.744 e^−09^	7.116 e^−08^	0.732	[0.51; 0.88]
Tilmicosin - ylosin	3.602	3.148 e^−04^	4.722 e^−03^	0.450	[0.17; 0.67]

Results of post-hoc pairwise comparisons following a significant global test (e.g., Kruskal-Wallis or ANOVA). *Z*-scores, unadjusted *p*-values (*P* unadj), and adjusted *p*-values (*P* adj) using the Bonferroni correction are shown for all treatment pairings. Significant differences (*P* adj < 0.05) indicate that the performance of the first-named treatment in each pair is significantly different from that of the second.

While significant differences in MIC magnitude were observed between *Spirulina platensis* and conventional antibiotics, with higher MIC values indicating lower *in vitro* potency for *Spirulina*, its potential advantages may relate to factors other than potency, such as its distinct activity profile or reported safety characteristics. Specifically, *Spirulina platensis* showed highly significant differences compared to doxycycline (p.adj < 0.001, rᵣᵦ = −0.95, 95% CI [−0.97, −0.92]), oxytetracycline (p.adj < 0.001, rᵣᵦ = −0.88, 95% CI [−0.92, −0.82]) and Aivlosin (p.adj < 0.001, rᵣᵦ = −0.91, 95% CI [−0.94, −0.86]).

Significant differences with moderate to large effect sizes were also observed between several conventional antibiotics. In fact, doxycycline demonstrated significantly lower MICs than oxytetracycline (p.adj < 0.001, rᵣᵦ = −0.55, 95% CI [−0.65, −0.42]), while tilmicosin and tylosin were found to have distinct activity levels (p.adj < 0.01, rᵣᵦ = 0.45, 95% CI [0.20, 0.65]).

Overall, these results indicate that the antimicrobial agents tested, including *Spirulina platensis*, differed significantly in their activity against *M. gallisepticum*. The distinct profile of *Spirulina platensis*, characterized by its significant separation from conventional antibiotics, supports its role as a unique natural alternative worthy of further investigation (Table 3).

### 3.5 Phenotypic profiling of antimicrobial response

#### 3.5.1 Hierarchical clustering of spirulina MIC profiles

**Figure 4:**
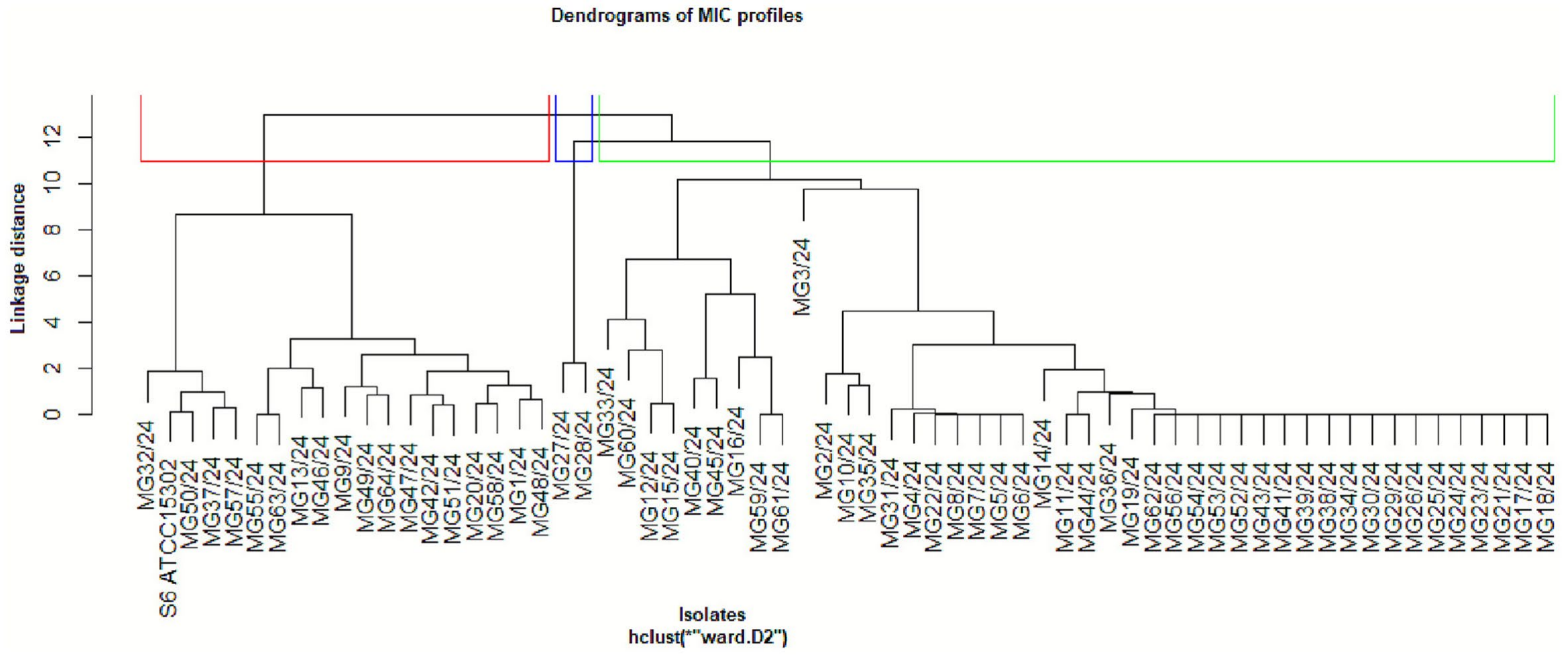
Hierarchical clustering of *Spirulina* MIC profiles. Dendrogram revealing MG clusters based on their MIC responses. Cluster 1 (*red*) includes susceptible strains with low MICs to both *Spirulina* and antibiotics, some of which exhibit selective resistance to *Spirulina*. Cluster 2 (*blue*) shows moderate-to-high MICs for *Spirulina* and elevated antibiotic resistance. Cluster 3 (g*reen*) comprises macrolide-resistants isolates with high MICs for multiple antibiotics (e.g., doxycycline at 0.06 μg/mL, tilmicosin at 32 μg/mL) and sensitive to *Spirulina platensis*.

Based on MIC values, hierarchical clustering analysis revealed distinct resistance patterns among MG strains, highlighting the potential of *Spirulina platensis* as a complementary or exploratory antimicrobial agent. Using complete linkage clustering, the resulting dendrogram identified three major clusters representing different susceptibility profiles (Figure 4). Cluster 3 consisted of macrolide-resistant strains which, while exhibiting high resistance to doxycycline (MIC = 0.06 μg/mL) and tilmicosin (MIC = 32 μg/mL), remained sensitive to *Spirulina platensis*, indicating that it acts through distinct mechanisms than those targeted by conventional antimicrobials. In contrast, Cluster 1 included strains that were generally susceptible to antibiotics but less responsive to *Spirulina*, while Cluster 2 comprised isolates with intermediate resistance patterns. The distinct separation of *Spirulina*’s activity from that of conventional agents emphasizes its therapeutic value, particularly against MDR strains. These findings support further exploration of *Spirulina platensis* as a novel, biologically based treatment option for resistant *Mycoplasma* infections.

#### 3.5.2 Heatmap analysis of antimicrobial susceptibility patterns

**Figure 5:**
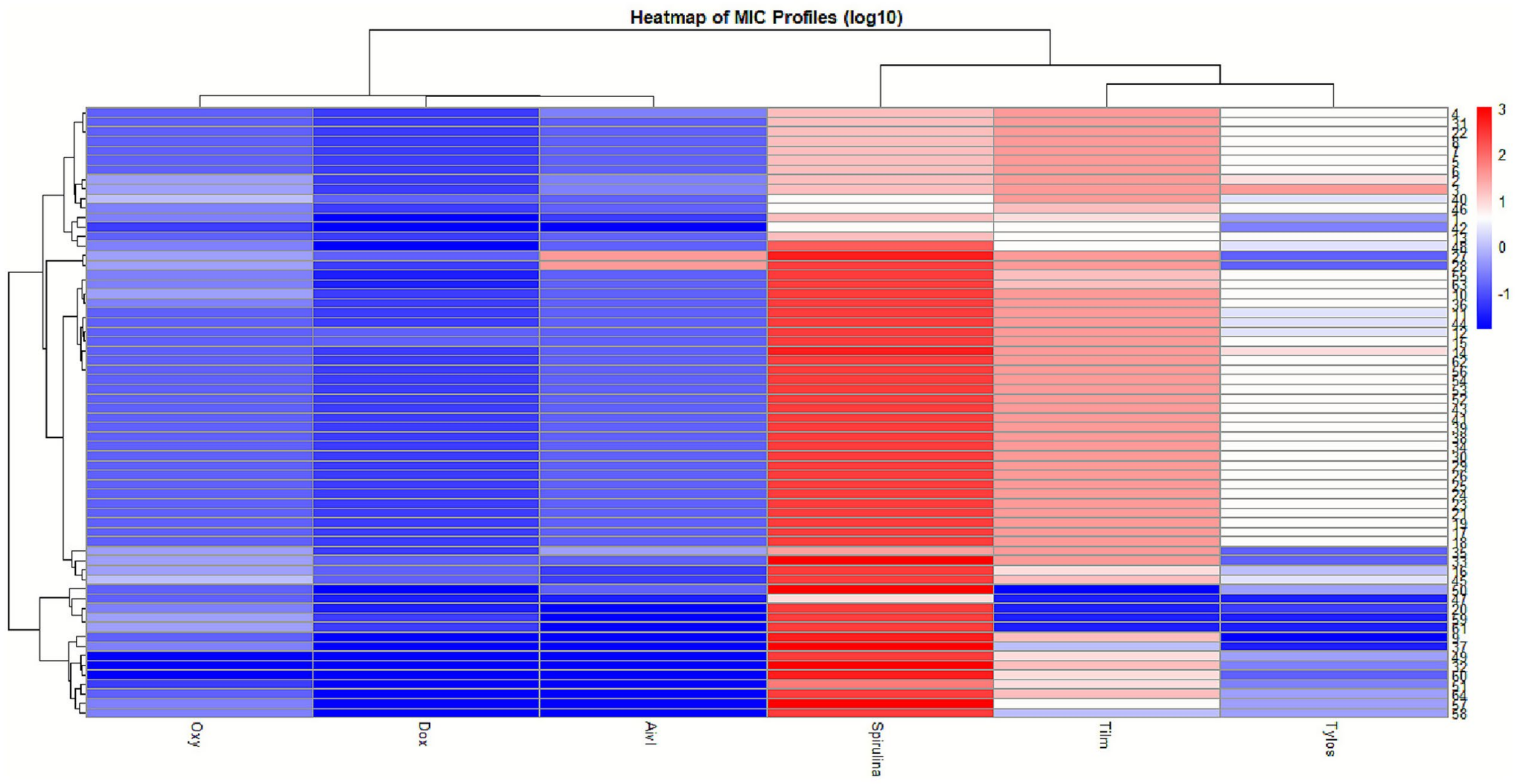
Heatmap and hierarchical clustering of antimicrobial susceptibility profiles in *Mycoplasma gallisepticum*. The heatmap displays log_10_-transformed MIC values (μg/mL) for 64 isolates tested against two tetracyclines (DOX, OXY), two macrolides (TIL, TYL), Aivlosin (AIV), and a *Spirulina platensis* extract. The color scale from blue (low MIC) to red (high MIC) represents a gradient of activity. Clustering analysis groups the *Spirulina platensis* extract with the tetracycline antibiotics, indicating correlated MIC profiles among the isolates.

The heatmap displays log₁₀-transformed MIC values for 64 MG isolates tested against six antimicrobial agents (Figure 5). Each row represents a single isolate, with the color gradient indicating MIC levels: darker blue for the lowest MICs and red for the highest MICs. Hierarchical clustering analysis, visualized by dendrograms, grouped agents and isolates based on MIC profile similarity. As expected, doxycycline (DOX) and oxytetracycline (OXY) formed a tight cluster, confirming their related mechanism of action. Notably, the *Spirulina platensis* extract (SPI) co-clustered with the tetracyclines, indicating a correlated MIC profile. The SPI column showed a gradient from light green/yellow to orange/red, representing a spectrum of moderate to higher MIC values among the isolates, with none exhibiting the darkest blue tones associated with the lowest MICs in this assay. This pattern indicates that, while the absolute MICs for SPI are generally higher than those of the most potent conventional antibiotics tested, its activity across isolates is structured and correlates with the tetracycline susceptibility phenotype. This correlation suggests a strain-dependent response and a potential shared susceptibility determinant with tetracyclines.

### 3.6 Bivariate correlation analysis

**Figure 6:**
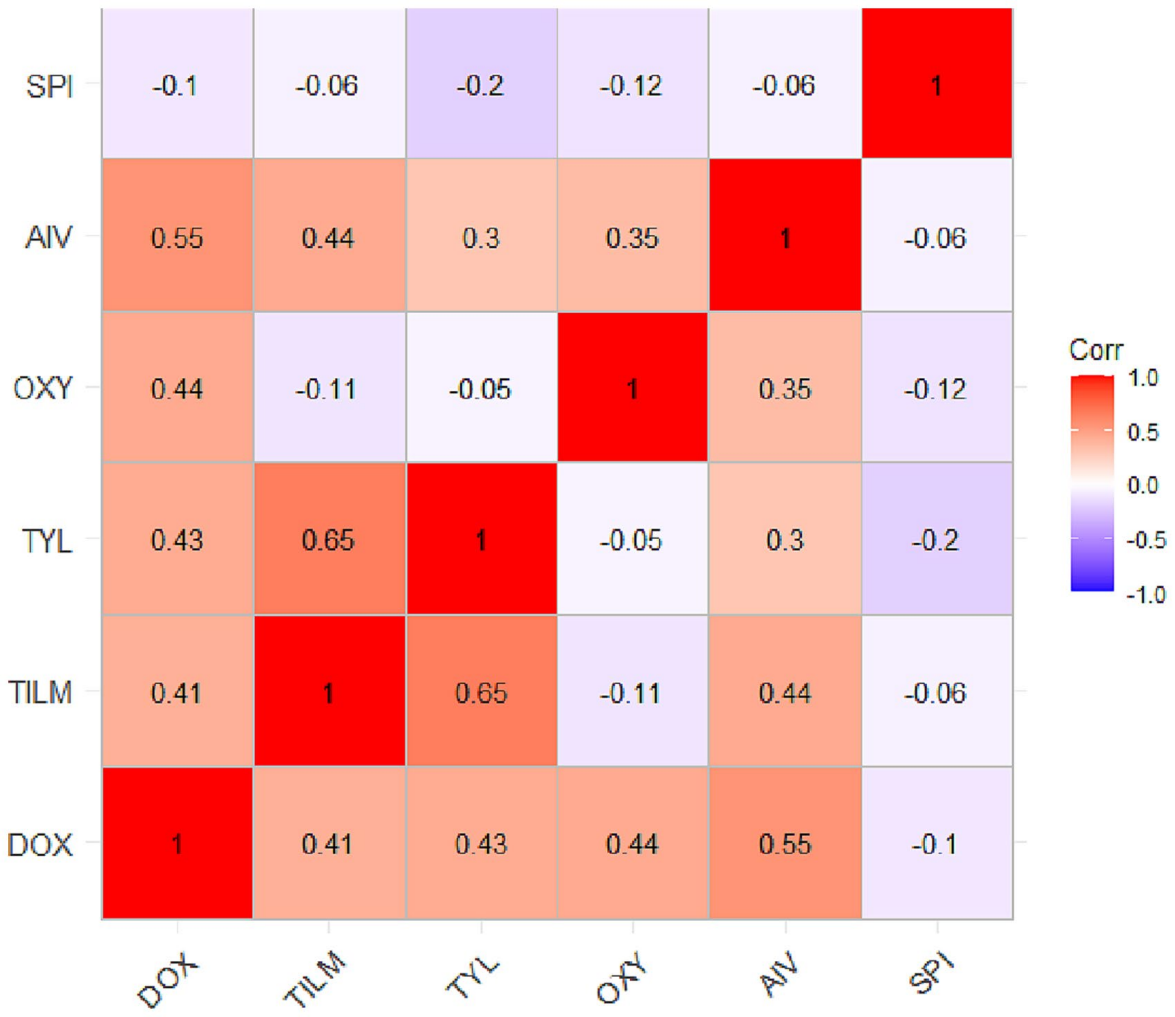
Pearson correlation matrix of antimicrobial MIC values among MG isolates. Correlation heatmap displaying Pearson correlation coefficients between MIC values of doxycycline (DOX), oxytetracycline (OXY), tilmicosin (TIL), tylosin (TYL), Aivlosin (AIV), and *Spirulina platensis* extract (SPI). Color scale ranges from blue (negative correlation) through white (no correlation) to red (positive correlation). Correlation coefficients are displayed within each cell.

Associations between the MIC values (log₁₀-transformed) of the tested antimicrobial agents were assessed. Given the non-normal distribution of the data, both Pearson and Spearman rank correlation analyses were performed. The results revealed consistent patterns between the two methods for most comparisons (Figure 6; Table 4).

**Table 4:** Comparison of Pearson and Spearman correlation coefficients for antimicrobial MIC values (log₁₀-transformed) from 64 *Mycoplasma gallisepticum* isolates.

Comparison	Pearson_*r*	Pearson_95%CI	Pearson_*p*	Spearman_rho	Spearman_*p*	Interpretation
DOX vs. TIL	0.409	[0.181, 0.595]	0.001	0.649	0	Very strong agreement
DOX vs. TYL	0.433	[0.209, 0.613]	0	0.365	0.003	Strong agreement
DOX vs. OXY	0.438	[0.216, 0.618]	0	0.297	0.017	Strong agreement
DOX vs. AIV	0.554	[0.357, 0.704]	0	0.568	0	Very strong agreement
DOX vs. SPI	0.176	[−0.073, 0.404]	0.165	0.199	0.115	Both non-significant
TIL vs. TYL	0.647	[0.477, 0.770]	0	0.661	0	Very strong agreement
TIL vs. OXY	−0.108	[−0.345, 0.142]	0.396	−0.122	0.338	Both non-significant
TIL vs. AIV	0.445	[0.224, 0.623]	0	0.752	0	Very strong agreement
TIL vs. SPI	0.158	[−0.091, 0.389]	0.212	0.146	0.249	Both non-significant
TYL vs. OXY	−0.052	[−0.294, 0.196]	0.683	−0.169	0.183	Both non-significant
TYL vs. AIV	0.297	[0.055, 0.506]	0.017	0.594	0	Strong agreement
TYL vs. SPI	−0.009	[−0.255, 0.237]	0.941	−0.025	0.845	Both non-significant
OXY vs. AIV	0.351	[0.115, 0.549]	0.005	0.187	0.139	Pearson only significant
OXY vs. SPI	0.087	[−0.162, 0.326]	0.494	0.120	0.345	Both non-significant
AIV vs. SPI	0.111	[−0.138, 0.347]	0.382	−0.016	0.901	Both non-significant

Comparison of Pearson and Spearman correlation coefficients for antimicrobial MIC values. Shown is the pairwise associations between the log₁₀-transformed MIC values of six antimicrobial agents tested against 64 *Mycoplasma gallisepticum* isolates. For each pair, Pearson’s correlation coefficient (*r*), its 95% confidence interval (CI), and *p*-value are shown alongside Spearman’s rank correlation coefficient (ρ) and its *p*-value. The final column provides an interpretation of the agreement between the two methods.

A strong, significant positive association was confirmed between tilmicosin and tylosin (Pearson’s *r* = 0.647, 95% CI [0.477, 0.770]; Spearman’s *ρ* = 0.661, *p* < 0.001), consistent with their shared macrolide class. A moderate positive association was also observed between doxycycline and Aivlosin (Pearson’s *r* = 0.554, 95% CI [0.357, 0.704]; Spearman’s ρ = 0.568, *p* < 0.001), suggesting potential cross-resistance.

In contrast, *Spirulina platensis* showed negligible or weak correlations with conventional antibiotics. Most associations were non-significant in both analyses. For example, the correlation between *Spirulina platensis* and tylosin was negligible (Pearson’s *r* = −0.009, 95% CI [−0.255, 0.237]; Spearman’s ρ = −0.025, *p* = 0.845). The strong concordance between Pearson and Spearman coefficients for key antibiotic pairs (e.g., tilmicosin-tylosin, doxycycline-Aivlosin) confirms that the observed correlation structure is robust and not an artifact of distributional assumptions (Table 4).

### 3.7 Principal component analysis

**Figure 7:**
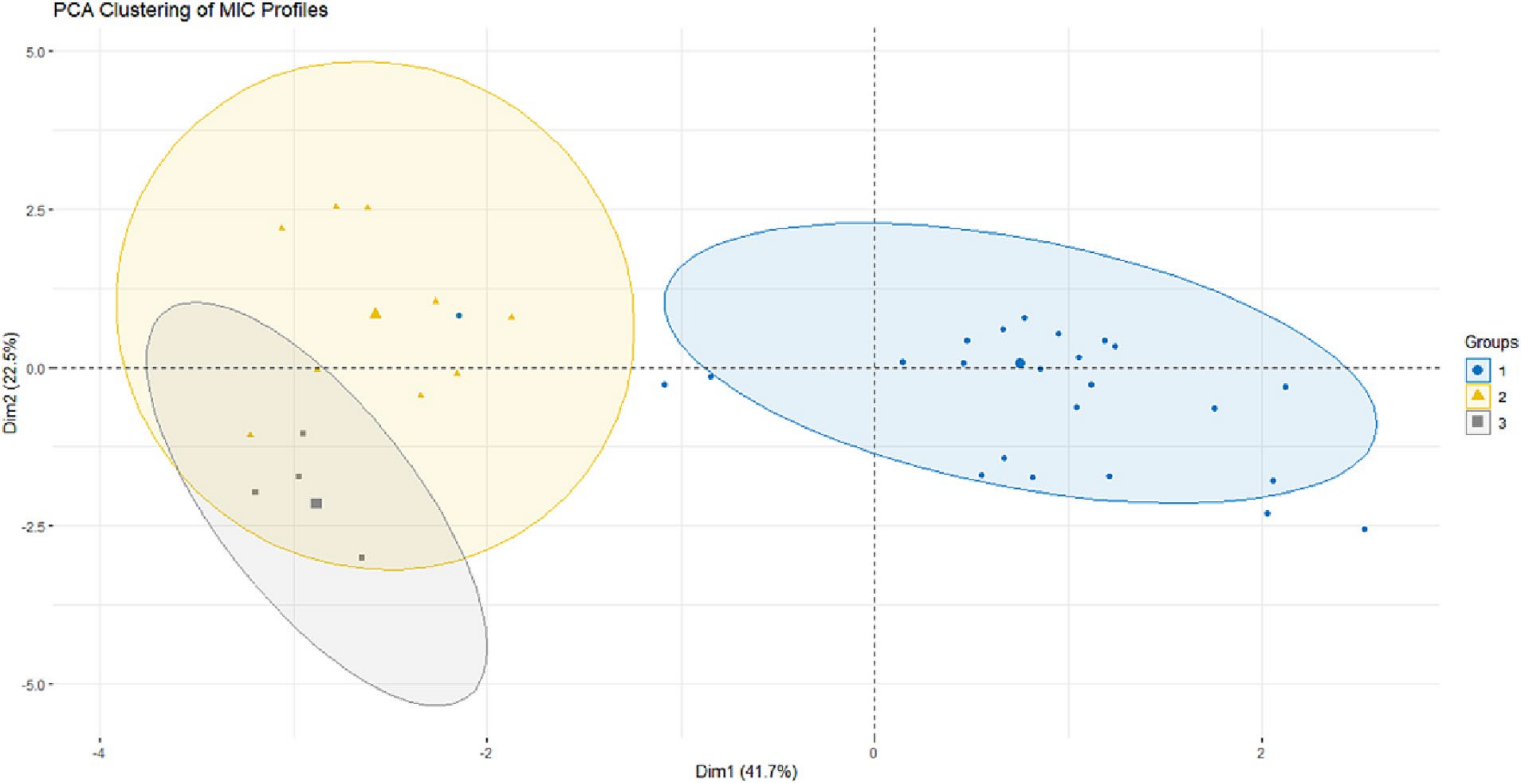
PCA biplot of antimicrobial susceptibility profiles among MG. The biplot illustrates the distribution of isolates based on their MIC profiles for six antimicrobial agents: doxycycline (DOX), oxytetracycline (OXY), tilmicosin (TIL), tylosin (TYL), Aivlosin (AIV), and a *Spirulina platensis* extract (SPI). Principal component 1 (Dim1) explains 41.75% of the total variance, and principal component 2 (Dim2) explains 12.25%. Isolates are colored and shaped according to clusters previously defined by hierarchical clustering of MIC data (see Figure 3): Group 1 (blue circles), Group 2 (yellow triangles), and Group 3 (grey squares). The PCA visualization confirms the separation of these susceptibility-based groups within the multivariate space.

**Figure 8:**
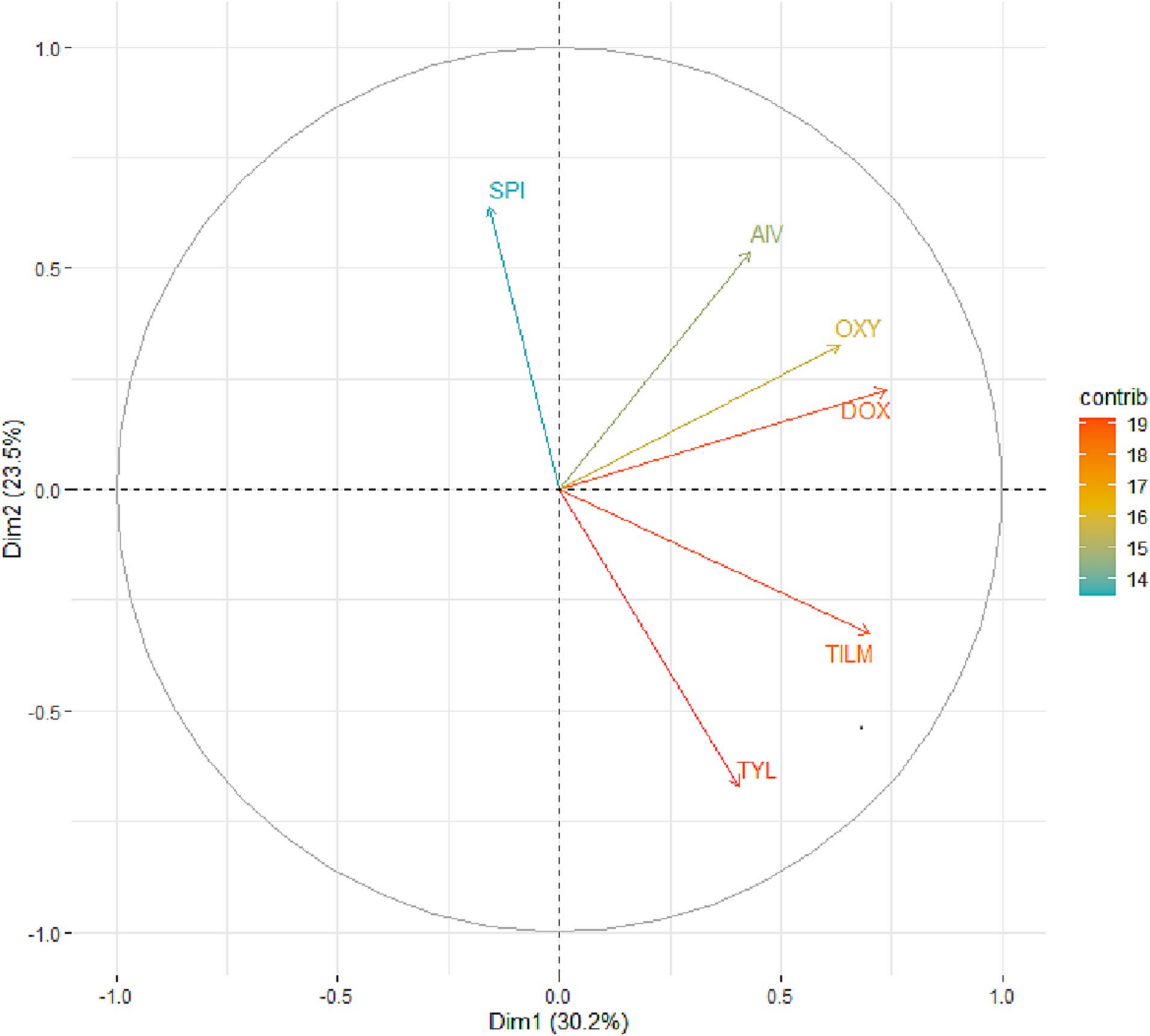
Relationships between *Spirulina* and other antibiotics. PCA correlation circle displaying the relationships between antimicrobial agents based on their MIC profiles across MG. Vectors represent doxycycline (DOX), oxytetracycline (OXY), tilmicosin (TIL), tylosin (TYL), Aivlosin (AIV), and *Spirulina platensis* extract (SPI) treatments. Vector length indicates the strength of contribution to the principal components, while angles between vectors reflect correlations. Vectors pointing in similar directions indicate positive correlations, while perpendicular vectors suggest independence.

The PCA was performed to explore phenotypic variability in antimicrobial susceptibility among macrolide-resistant MG isolates. The PCA biplot of MIC data revealed three distinct clusters, each reflecting specific antimicrobial response patterns (Figure 7). Group 1 included isolates with low MICs across all treatments, indicating high susceptibility. Conversely, Groups 2 and 3 contained isolates with moderate and high MICs, respectively, likely representing macrolide-resistant profiles. The relationships among the antimicrobial agents, illustrated in the PCA correlation circle (Figure 8), are shown in Figure 8. *Spirulina platensis* was positioned orthogonally to tylosin and tilmicosin, indicating statistical independence (*r* ≈ 0) and suggesting a mechanism different from that of conventional antibiotics. In contrast, doxycycline, oxytetracycline, Aivlosin, and tilmicosin clustered closely together, forming inter-vector angles of less than 45°, indicating strong positive correlations (*r* > 0.7, *p* < 0.001) and suggesting potential shared resistance mechanisms.

**Figure 9:**
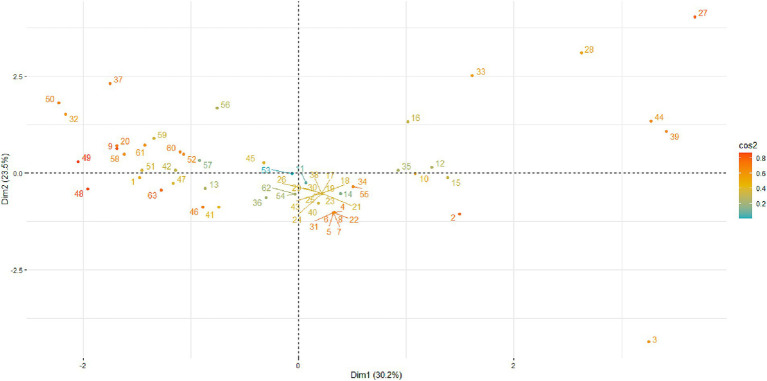
PCA individual factor map of MG isolates. PCA scatter plot showing the distribution of 64 MG isolates in two-dimensional space based on their antimicrobial susceptibility profiles. Each point represents an individual isolate, with color intensity reflecting the cos^2^ values (quality of representation) for each isolate on the first two principal components; darker colors indicate higher cos^2^ values (*better representation by PC1 and PC2*).

The individuals’ factor map shows the distribution of isolates in the PCA space (Figure 9), with color intensity indicating cos^2^ values, which represent the degree of contribution of the first two principal components of each isolate. Isolates positioned at the edge of the plot, especially isolates #3, #27, and #28, contributed most significantly to the overall variance and therefore correspond to highly resistant or phenotypically distinct strains. Additionally, Dimension 1 accounted for 30.2% of the variance, while Dimension 2 explained 23.4%, highlighting the complexity and diversity of resistance profiles within the population. Overall, these findings highlight the distinct antimicrobial activity of *Spirulina platensis* and support its potential as a complementary or exploratory approach in the management of *Mycoplasma gallisepticum* infections, rather than as an alternative to conventional antibiotics.

#### 3.7.1 Predictive modeling of spirulina susceptibility using conventional antibiotic profiles

**Figure 10:**
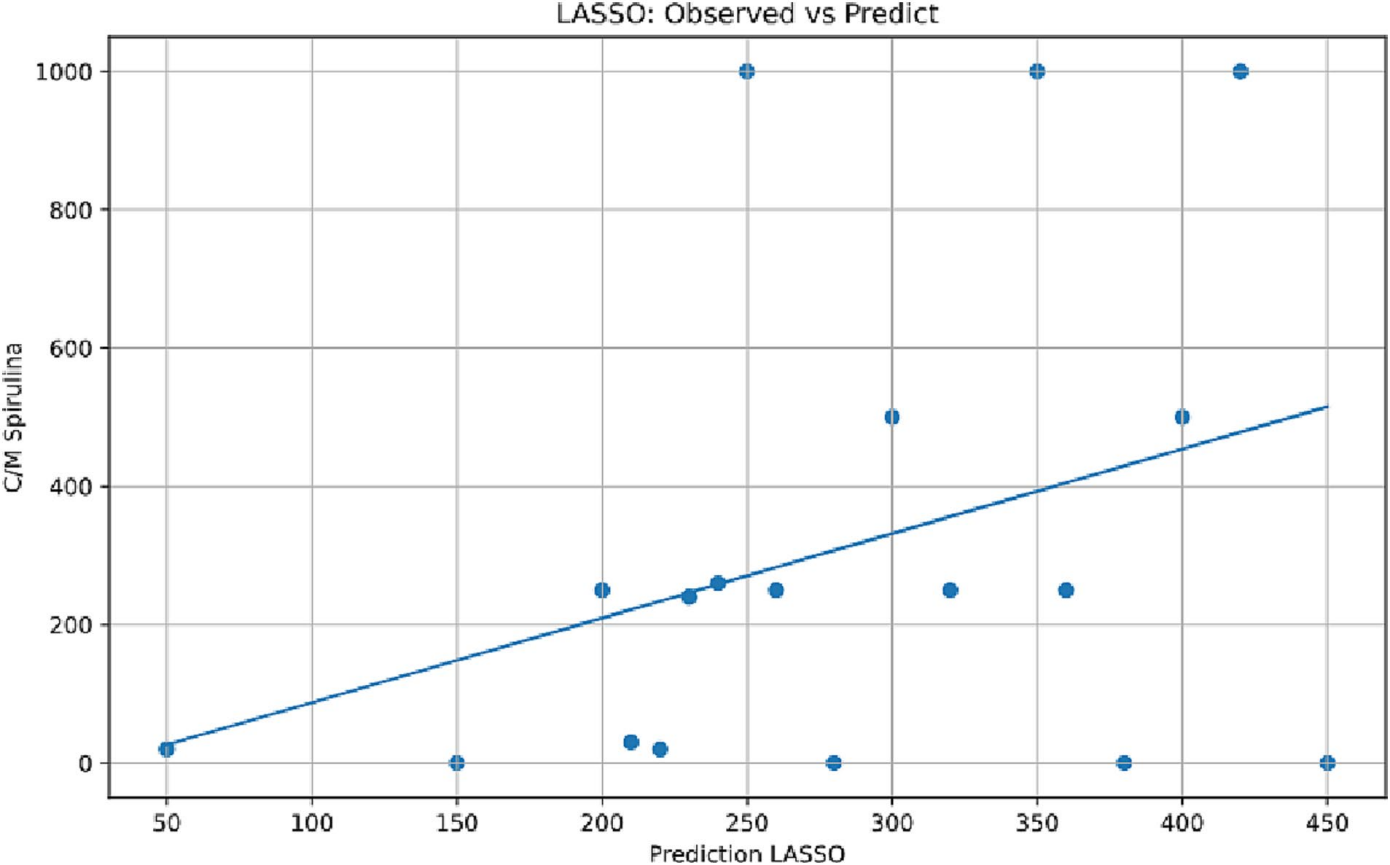
LASSO regression model performance for *Spirulina* MIC prediction. Scatter plot comparing observed versus predicted *Spirulina platensis* MIC values using LASSO (least absolute shrinkage and selection operator) regression based on conventional antibiotic MIC profiles. Each point represents an individual MG isolate, with the diagonal dashed line indicating perfect prediction (1:1 correspondence).

**Figure 11:**
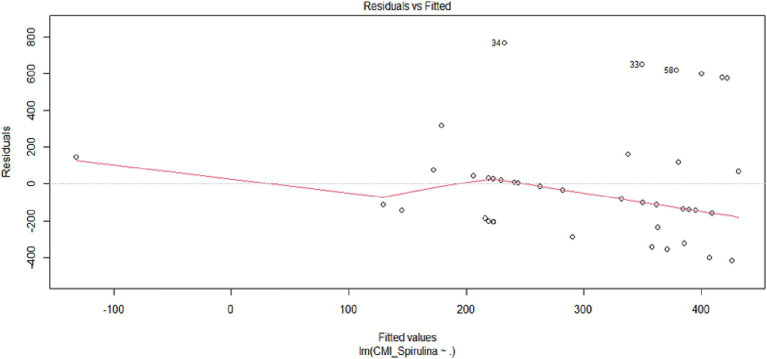
Multiple linear regression diagnostic plot for *Spirulina* MIC prediction. Residuals versus fitted values plot from a multiple linear regression model predicting *Spirulina platensis* MIC values based on antibiotic MIC profiles. Each point represents an individual MG isolate, with residuals (observed minus predicted values) plotted against fitted (predicted) values. The horizontal dashed line at zero indicates perfect prediction. Diagnostic analysis uncovers violations of key regression assumptions: (1) Non-random residual distribution with downward curvature suggesting non-linearity; (2) Increasing residual spread at higher fitted values indicating heteroscedasticity; and (3) Influential outliers including isolates #34, #33, #58, and #60 with large residuals.

LASSO (Least Absolute Shrinkage and Selection Operator; Figure 10) and linear regression (Figure 11) models were used to assess whether susceptibility to conventional antibiotics can predict MG responses to *Spirulina platensis*. While the LASSO regression model showed a moderate correlation between predicted and observed *Spirulina* MIC values (Figure 8), isolates with high MICs (≥500 μg/mL) were consistently underpredicted, suggesting that the model may have failed to capture complex or nonlinear resistance dynamics. The narrow range of predicted values (200–400 μg/mL) indicates that key predictors influencing high-level resistance were either missing or poorly represented in the model.

Further evaluation of the multiple linear regression model revealed deviations from key statistical assumptions (Figure 11). Residual plots exhibited curvature and heteroscedasticity, particularly at higher fitted MIC values, indicating a poor model fit. Outliers, specifically isolates #33, #34, #58, and #60, produced large residuals, possibly due to atypical or macrolide-resistant phenotypes not accounted for by standard antibiotic MIC predictors. This suggests that conventional regression methods have limited usefulness in predicting susceptibility to *Spirulina platensis*, supporting the notion that *Spirulina platensis* exerts its effects through mechanisms distinct from those of common antibiotics.

**Figure 12:**
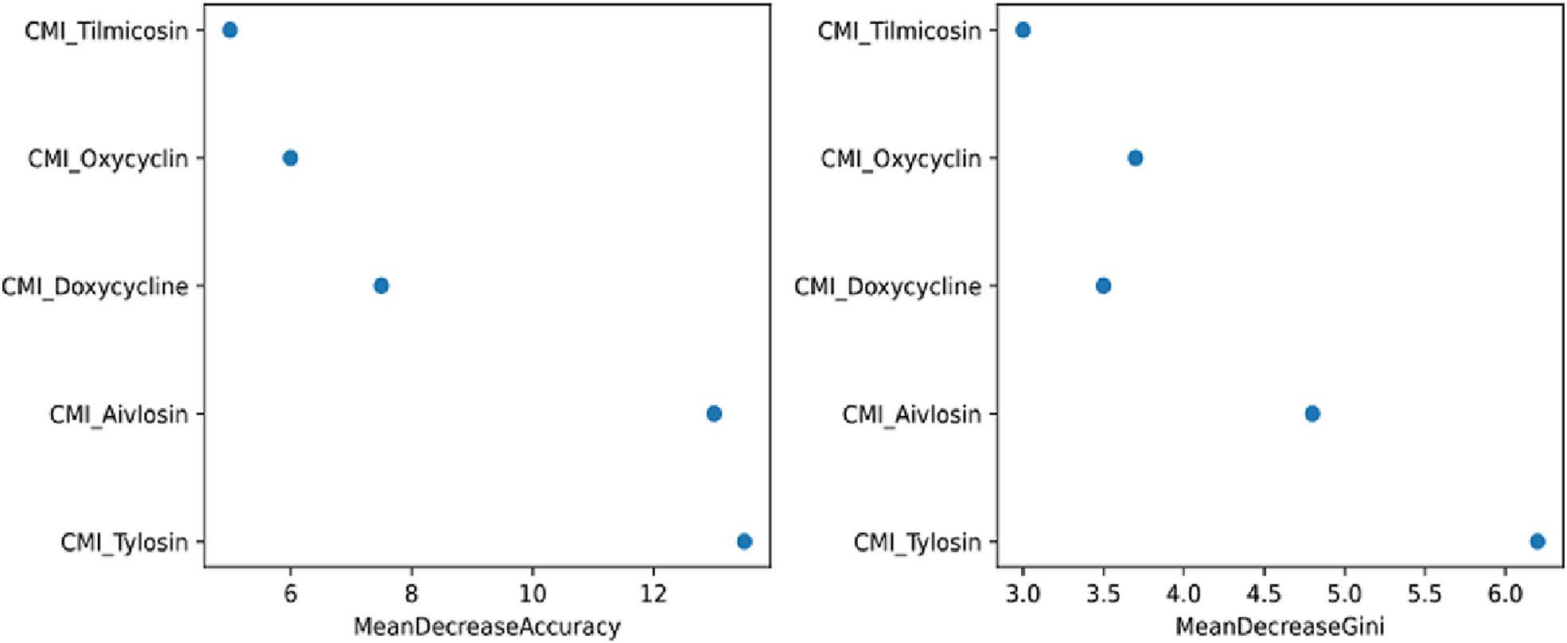
Random forest variable importance analysis for *Spirulina* MIC prediction variable importance plot from random forest model showing the relative contribution of conventional antibiotic MIC values in predicting *Spirulina platensis* susceptibility among MG. Displayed are: mean decrease accuracy measuring the decrease in model accuracy when each variable is randomly permuted (*blue bars*), and mean decrease Gini measuring the average decrease in node impurity when each variable is used for splitting (*red bars*).

Random Forest (RF) analysis, based on both Mean Decrease in Accuracy and Mean Decrease in Gini metrics, identified tylosin as the most important predictor of *Spirulina* MIC, followed by Aivlosin and doxycycline (Figure 12). Although *Spirulina platensis* originates from a fundamentally different biological source, the consistent predictive value of macrolide and tetracycline MICs suggests the existence of overlapping phenotypic resistance traits. These findings imply that, despite *Spirulina*’s unique mechanism of action, susceptibility to it may still be influenced by resistance profiles typically associated with conventional antimicrobials. This highlights a potential convergence in microbial response pathways and warrants further investigation into possible cross-resistance mechanisms or shared molecular targets.

### 3.8 Evaluation of *Spirulina platensis* biocompatibility

The cytotoxicity of the *Spirulina platensis* extract was assessed on RAW 264.7 cells using the MTT assay. Data are presented as the mean ± standard deviation of three independent biological replicates, each performed with four technical replicates. Over the tested concentration range (125 to 4,000 μg/mL), the extract showed no significant cytotoxic effect, with cell viability consistently exceeding 90% at all concentrations compared to the untreated control (*p* > 0.05, one-way ANOVA) (Table 5). The effect size for the overall ANOVA was negligible (η^2^ < 0.01). A concentration of 2,000 μg/mL was thus established as a reliable non-toxic threshold in this model.

**Table 5:** Antimicrobial, cytotoxicity, and selectivity index for local MG isolates.

Isolates	CMIa *Spirulina platensis* (μg/mL)	CMIc *Spirulina platensis* (μg/mL)	SI
S6 ATCC15302	**1,000**	**2000**	**2**
MG1/24	15.62	2000	128.04
MG2/24	15.62	2000	128.04
MG3/24	15.62	2000	128.04
MG4/24	15.62	2000	128.04
MG5/24	15.62	2000	128.04
MG6/24	15.62	2000	128.04
MG7/24	15.62	2000	128.04
MG8/24	15.62	2000	128.04
MG9/24	500	2000	4
MG10/24	250	2000	8
MG11/24	250	2000	8
MG12/24	250	2000	8
MG13/24	15.62	2000	128.04
MG14/24	500	2000	4
MG15/24	250	2000	8
MG16/24	250	2000	8
MG17/24	250	2000	8
MG18/24	250	2000	8
MG19/24	250	2000	8
MG20/24	250	2000	8
MG21/24	250	2000	8
MG22/24	15.62	2000	128.04
MG23/24	250	2000	8
MG24/24	250	2000	8
MG25/24	250	2000	8
MG26/24	250	2000	8
MG27/24	500	2000	8
MG28/24	250	2000	8
MG29/24	250	2000	8
MG30/24	250	2000	8
MG31/24	15.62	2000	128.04
MG32/24	1,000	2000	2
MG33/24	1,000	2000	2
MG34/24	250	2000	8
MG35/24	31.25	2000	64
MG36/24	250	2000	8
MG37/24	1,000	2000	2
MG38/24	250	2000	8
MG39/24	250	2000	8
MG40/24	3.9	2000	512.82
MG41/24	250	2000	8
MG42/24	3.9	2000	512.82
MG43/24	250	2000	8
MG44/24	250	2000	8
MG45/24	250	2000	8
MG46/24	3.9	2000	512.82
MG47/24	7.81	2000	256.08
MG48/24	125	2000	16
MG49/24	250	2000	8
MG50/24	1,000	2000	2
MG51/24	62.5	2000	32
MG52/24	250	2000	8
MG53/24	250	2000	8
MG54/24	250	2000	8
MG55/24	250	2000	8
MG56/24	250	2000	8
MG57/24	1,000	2000	2
MG58/24	250	2000	8
MG59/24	250	2000	8
MG60/24	500	2000	8
MG61/24	250	2000	8
MG62/24	250	2000	8
MG63/24	250	2000	8
MG64/24	250	2000	8

CMIa, antimicrobial activity of *Spirulina platensis*; CMIc, cytotoxicity of *Spirulina platensis*; SI, selectivity index.

*In vitro* assessment of the Selectivity Index (SI) for *Spirulina platensis* extracts against *Mycoplasma gallisepticum* isolates, including reference strain S6 ATCC15302. CMIa represents the minimum inhibitory concentration activity (MIC, μg/mL) of the *Spirulina platensis* extract against the MG isolates. CMIc represents the minimum inhibitory concentration cytotoxicity (μg/mL) against host cells. The Selectivity Index (SI) was calculated as follows: SI = CMIc/CMIa. A higher SI value indicates a wider safety margin and greater therapeutic potential. The bold values correspond to the reference strain (ATCC).

The calculated selectivity index (SI) values, which ranged from 2 to 512.82 across the 64 *Mycoplasma gallisepticum* isolates, indicated considerable variation in the therapeutic window. A *post hoc* power analysis for the key comparison of cell viability at 4000 μg/mL versus the control, based on the observed effect size and variance, confirmed a statistical power > 80% (alpha = 0.05), supporting the robustness of the non-toxicity conclusion (Table 5).

These results confirm the high biocompatibility of *Spirulina platensis* and its safety at concentrations relevant for antimicrobial testing. The absence of cytotoxicity supports its use in subsequent antibacterial evaluations and underscores its potential as a safe, natural antimicrobial agent.

## 4 Discussion

Poultry production is the most common form of animal farming worldwide and plays a vital role in ensuring food security, especially in low- and middle-income countries. In 2020, chicken meat accounted for about 35% of total global meat production, underscoring its importance to human nutrition and food systems (Gilbert et al., 2018). However, this essential sector faces ongoing threats from infectious diseases, particularly MG, the most pathogenic avian mycoplasma. MG infections cause significant economic losses by reducing egg production, lowering hatchability, impairing carcass quality, and increasing flock mortality (Armour, 2020). Despite the widespread use of vaccination programs and biosecurity measures (Kleven, 2008), MG remains endemic in many regions due to its high genetic variability, ability to evade the immune system, and multiple transmission routes (Fang et al., 2025; Butenko et al., 2017). This underscores the urgent need for innovative and sustainable control strategies.

Given the increasing threat of antimicrobial resistance (AMR) in animal agriculture, exploring alternatives to traditional antibiotics is both timely and necessary (Kasimanickam et al., 2021). MG is a key poultry pathogen and is increasingly resistant to standard antimicrobials, thereby endangering flock health and food security. While antibiotics remain the primary method for controlling outbreaks, the rising incidence of AMR in MG strains is undermining treatment outcomes (Kasimanickam et al., 2021; Elbehiry et al., 2025). Our study identified a concerning level of macrolide resistance, especially to tilmicosin (87.5%), tylosin (68.75%), and Aivlosin (9.37%); 46.87% of strains developed resistance to two or more, and an alarming third exhibited resistance to all three macrolides. This further confirms previous reports of widespread resistance and antibiotic overuse in veterinary medicine (Chniba et al., 2020; Elbehiry et al., 2025; Huang et al., 2021).

Our findings indicate that local MG isolates from Tunisia have notably higher MICs than those reported in neighboring regions, such as Egypt (Abd El-Hamid et al., 2019; Emam et al., 2020). This difference indicates significant geographic and strain-specific variation in antimicrobial susceptibility, likely influenced by local antibiotic use and selection pressures (Kasimanickam et al., 2021; Ma et al., 2023). Molecular studies have linked macrolide resistance to specific mutations in the 23S rRNA gene (A2058G) and the ribosomal protein gene rplD (Arg177Lys), which impair drug binding and lead to therapeutic failure (Chniba et al., 2020; Gerchman et al., 2011). These results highlight the complex relationship between genetic adaptation and resistance development, underscoring the urgent need for innovative, non-antibiotic treatments to control MG infections and reduce antimicrobial resistance in poultry production (Bomblies and Peichel, 2022).

The nutrient-dense cyanobacterium *Spirulina platensis* is increasingly recognized for its multifunctional antimicrobial, antioxidant, and immunomodulatory properties. It has shown protective benefits in poultry production, such as improved gut integrity during coccidiosis (Fries-Craft et al., 2021), reduction of organ damage caused by mycotoxins (Manafi, 2011), and boosted immune responses (An et al., 2020). Our study confirms the antibacterial efficacy of *S. platensis* against MG, with MICs ranging from 3.9 to 1,000 μg/mL, and more than half of the isolates were inhibited at concentrations ≤250 μg/mL. These findings align with earlier research indicating *Spirulina*’s superior antimicrobial activity compared to other algal species (Elshouny et al., 2021). Extracts rich in phenolic compounds, especially those prepared with methanol or DMSO, have shown significant inhibition of *Pseudomonas aeruginosa*, *Staphylococcus aureus*, and *Bacillus subtilis* (Gheda and Ismail, 2020; El-Sheekh et al., 2014), emphasizing their potential as broad-spectrum antimicrobial agents in veterinary use.

Several mechanisms have been proposed to explain the antimicrobial effects of *Spirulina platensis*, including disruption of bacterial membranes, interference with quorum sensing and motility, and inhibition of biofilm formation (Abd El-Hack et al., 2020; Bou-Kassem et al., 2021; Saleh et al., 2021). These pathways resemble those of plant-derived compounds, which have been shown to inhibit *Campylobacter jejuni* biofilm formation and quorum sensing by 75–90% (Alum et al., 2025). In our study, *Spirulina platensis* demonstrated strong activity against macrolide-resistant MG strains, suggesting a likely synergistic effect among its bioactive compounds, including phycocyanin, carotenoids, alkaloids, and terpenoids (El-Shall et al., 2023). Compared to essential oils and plant extracts, *Spirulina platensis* demonstrated measurable *in vitro* antimicrobial activity; however, its higher MIC values indicate lower potency, and any potential applications should be interpreted independently of efficacy claims (Shah et al., 2024).

Taken together, these findings highlight the need for further research into the molecular interactions of *S. platensis* and its incorporation into antimicrobial strategies, especially considering the increasing resistance to conventional drugs (An et al., 2020; Metekia and Ulusoy, 2023). The observed inhibitory effects suggest that *Spirulina platensis* is a practical, natural alternative for managing MG infections, promoting a more sustainable and responsible use of antimicrobials in poultry health management.

Cytotoxicity testing confirmed the high biocompatibility of *Spirulina platensis*, with no significant reduction in cell viability at all the tested concentrations up to 4,000 μg/mL. This was comparable to that of some essential oils, which have shown route-dependent toxicity (Greff et al., 2023). DMSO proved effective in extracting and delivering *Spirulina*’s active compounds in a bioavailable form (Marjanović et al., 2024). The low MIC values obtained with DMSO-based extracts align with those previously reported for other bacterial species, confirming its suitability for antimicrobial testing. Our MIC values are comparable to plant-derived antimicrobials effective against MG, including clove and cumin oils (MIC: 0.49–15.63 μg/mL) and cinnamon oil (MIC: 500 μg/mL) (Abd El-Hamid et al., 2019; Sleha et al., 2014). On the other hand, *Spirulina*’s broader compound profile and synergistic activity may offer advantages over single-compound essential oils.

Currently, 12 vaccines against MG are available, including inactivated vaccines (e.g., R strain) (Jacob et al., 2014), live-attenuated vaccines [F strain (Cummings and Kleven, 1986), ts-11 (Whithear et al., 1990), 6/85 (Abd-el-Motelib and Kleven, 1993), K strain (Ferguson-Noel and Williams, 2015), ts-304 (Kanci et al., 2018), MG 7 (Gates et al., 2008), Vaxsafe MG304 (Kanci Condello et al., 2024)], and genetically engineered vaccines [GT5 (Papazisi et al., 2002), Vectormune FP-MG (Zhang et al., 2010), ts-11 C3 (Muneta et al., 2008), CT5 (Gates et al., 2021)]. Vaccination remains the cornerstone of MG prevention, especially in commercial laying hens and breeder flocks. However, its limitations are notable; in fact, protection can be incomplete, latent or breakthrough infections may still occur via horizontal or vertical transmission, and implementation entails high operational costs for flock-wide administration and ongoing surveillance. Consequently, vaccination is ideally supported by stringent biosecurity and monitoring measures. In this context, *Spirulina platensis* emerges as a promising complementary strategy. Its biomass is globally mass-produced at low cost, with primary expenses limited to extraction and formulation. While initial production costs may exceed those of conventional antibiotics, *Spirulina* offers a non-antibiotic, residue-free alternative that avoids recurring vaccine program expenditures and aligns with AMR mitigation goals. Thus, *Spirulina* could alleviate some economic and practical constraints of current MG control, pending further *in vivo* validation and formulation optimization.

Understanding trends in resistance and alternative antimicrobial options is essential for effective poultry disease control (Kasimanickam et al., 2021; Elbehiry et al., 2025). The high MDR levels observed highlight the importance of routine resistance monitoring and updated treatment protocols. One limitation of our study is the lack of standardized clinical breakpoints for *Spirulina platensis*, which makes definitive interpretation of efficacy difficult (Woo et al., 2023). Furthermore, as only a subset of isolates was subjected to molecular resistance profiling, this necessitates a comprehensive genetic analysis. On the other hand, our study has several strengths. Antibiotic treatment history was available for all flocks, thereby enabling direct correlation with resistance patterns. Microdilution plates customized with farm-specific antibiotics improved clinical relevance. Additionally, hierarchical clustering facilitated the classification of resistance profiles and the identification of MDR subsets. These methods offered a solid, field-relevant assessment of susceptibility patterns.

## 5 Conclusion

This study highlights *Spirulina platensis* as a promising natural antimicrobial agent against MG, including MDR strains resistant to tylosin and tilmicosin. The wide range of observed MICs indicates significant phenotypic diversity, underscoring the need for strain-specific, evidence-based treatment strategies to manage poultry health. The broad-spectrum antimicrobial activity of *Spirulina platensis*, along with its distinct mechanism of action and non-cytotoxic nature, suggests its integration into antibiotic stewardship programs. Its potential use as a bioactive feed additive or adjunctive therapeutic approach may represent a sustainable strategy to reduce reliance on traditional antibiotics. Future studies should include molecular characterization of resistance, dose optimization, and *in vivo* trials to evaluate its relevance under field conditions.

## Data Availability

The original contributions presented in the study are included in the article/supplementary material, further inquiries can be directed to the corresponding author.

## Footnotes

1
https://edenlife.com.tn/
